# GPCR-Hippo Signaling in Cancer

**DOI:** 10.3390/cells8050426

**Published:** 2019-05-08

**Authors:** Jiaqian Luo, Fa-Xing Yu

**Affiliations:** Children’s Hospital and Institutes of Biomedical Sciences, Fudan University, Shanghai 200032, China; jqluo15@fudan.edu.cn

**Keywords:** G protein-coupled receptor, GPCR, Hippo pathway, YAP/TAZ, signal transduction, cancer, tumorigenesis, anti-cancer therapy

## Abstract

The Hippo signaling pathway is involved in tissue size regulation and tumorigenesis. Genetic deletion or aberrant expression of some Hippo pathway genes lead to enhanced cell proliferation, tumorigenesis, and cancer metastasis. Recently, multiple studies have identified a wide range of upstream regulators of the Hippo pathway, including mechanical cues and ligands of G protein-coupled receptors (GPCRs). Through the activation related G proteins and possibly rearrangements of actin cytoskeleton, GPCR signaling can potently modulate the phosphorylation states and activity of YAP and TAZ, two homologous oncogenic transcriptional co-activators, and major effectors of the Hippo pathway. Herein, we summarize the network, regulation, and functions of GPCR-Hippo signaling, and we will also discuss potential anti-cancer therapies targeting GPCR-YAP signaling.

## 1. The Hippo Signaling Network

The Hippo pathway is initially established in *Drosophila melanogaster* (fruit flies), following extensive genetic screens for tumor suppressors, and is highly conserved in mammals [1,2]. The Hippo pathway plays a crucial role in regulating cell survival, proliferation, differentiation, and organ size [1,2,3,4]. The core Hippo pathway in mammals can be represented by a kinase cascade consisting of Ste20-like kinases 1/2 (MST1/2), MAP kinase kinase kinase kinases (MAP4K1-7), Large tumor suppressor 1/2 (LATS1/2), Salvador 1 (SAV1, also known as WW45), MOB kinase activator 1A/B (MOB1A/B), Yes-association protein (YAP), and Transcriptional coactivator with PDZ-binding motif (TAZ, also known as WWTR1), TEA domain family members (TEAD1-4), and Vestigial-like family member 4 (VGLL4). Mechanistically, MST1/2 in complex with SAV1 phosphorylate and activate LATS1/2, and MAP4K proteins plays overlapping, yet non-redundant, roles in activating LATS1/2 [5]. LATS1/2 subsequently phosphorylate multiple serine residues of YAP (including S127 and S318) and TAZ (including S89 and S311). Phosphorylation of YAP/TAZ lead to 14-3-3 mediated cytoplasmic retention and ubiquitination-dependent proteasome degradation [6,7,8]. When upstream kinases are inactivated, dephosphorylated YAP/TAZ translocate into the nucleus, bind with TEAD1-4 and induce the expression of target genes such as connective tissue growth factor (CTFG) and cysteine-rich angiogenic inducer 61(CYR61) [7,9]. Without nuclear YAP/TAZ, TEAD1-4 interact with VGLL4, which may repress transcription of target genes [10,11]. The Hippo signaling output is dependent on transcriptional activity of YAP/TAZ and the latter is mainly inhibited by Hippo pathway kinases.

Several proteins may interpret and transmit physiological signals to core components of the Hippo pathway. The apical membrane-associated FERM-domain protein Neurofibromin 2 (NF2, also known as Merlin) is an activator of the Hippo pathway [12]. NF2 functions by forming a complex with Kidney and brain (KIBRA, also known as WWC1) to activate MST1/2 or recruiting LATS1/2 to plasma membrane for activation by MST1/2 [13]. KIBRA may activate LATS1/2 in a MST1/2-dependent or -independent manner [14]. AMOT family proteins (AMOTp130, AMOTL1, and AMOTL2) interact with YAP/TAZ and enhance cytoplasmic or junctional localization of YAP/TAZ, and LATS1/2 activity is also mildly induced by AMOT proteins, both lead to an inhibition of YAP/TAZ activity [15,16,17,18]. Ras association domain family (RASSF) proteins interact with MST1/2 or SAV1, and may mediate RAS signaling to the Hippo pathway [19,20,21]. Further studies are required to understand how these proteins serve as bridges linking mechanical or biochemical cues and Hippo pathway kinases.

To date, an array of environmental stimuli has been shown to regulate YAP/TAZ activity. Cell-cell contact is well-known to suppress YAP/TAZ activity by promoting LATS1/2 activation [7]. The mechanical force, such as stiffness of extracellular matrix (ECM), cell geometry and shear stress, regulates phosphorylation and subcellular localization of YAP/TAZ, and recently small GTPase RAP2 has been shown to mediate matrix stiffness signals to LATS1/2 [22,23]. Various stress signals, including oxidative stress, hypoxia, energy stress, endoplasmic reticulum (ER) stress, and osmotic stress modulate YAP/TAZ activity as well [24,25,26,27,28,29]. Moreover, G protein-coupled receptors (GPCRs) can mediate diverse diffusive signals to modulate Hippo pathway activity, the regulation of the Hippo pathway by GPCR signaling will be further discussed below [30,31,32].

## 2. The Hippo Pathway in Tumorigenesis

The link between the Hippo pathway and cancer development has been recently reviewed elsewhere [33,34]. Among components of the Hippo pathway, YAP and TAZ are considered as oncoproteins, whereas most upstream regulators are with tumor suppressor functions. In mouse models, transgenic expression of *Yap*, or genetic ablation of *Nf2, Sav1, Mst1/2, Lats1/2, Mob1, Wwc1/2*, and *Rassf1a* all lead to tumorigenesis [35,36,37,38,39,40,41,42,43,44].

As oncoproteins, YAP/TAZ are able to promote cell proliferation, cell transformation, and cancer cell stemness. YAP/TAZ can induce cell proliferation and reduce cell death, which together lead to increased cell numbers [8,45]. YAP/TAZ may also promote cell transformation, as overexpression of YAP in human non-transformed mammary epithelial cells induces epithelial-to-mesenchymal transition (EMT), and increased TAZ expression in mammary cells leads to the acquisition of a spindle-shaped morphology and increased cell migration and invasion [46,47,48]. Recently, multiple studies have shown that YAP/TAZ play a role in regulating cancer stem cells (CSCs) [49]. YAP activation leads to dedifferentiate of matured cells and expands undifferentiated liver, epidermal, neural, cardiac, muscle, and intestinal stem/progenitor cells [37,50,51,52,53,54,55,56]. In breast cancer, TAZ expression is enriched in CSCs with high self-renewal and tumor initiating capacities [48]. YAP also induces esophageal CSC properties via upregulation of SOX9 [57]. Together, enhanced YAP/TAZ activity may promote cancer development by multiple approaches, such as modulating cell proliferation, movement, and stemness.

YAP/TAZ are activated in diverse human cancers and may serve as an indicator of poor prognosis. Elevated expression of YAP/TAZ is frequently observed in human cancers, including liver, breast, prostate, colorectal, gastric, lung, and brain tumors, especially in high-grade or metastatic tumors [56,58,59,60,61,62,63,64]. The expression of YAP/TAZ also functions as a prognostic marker, for instance, YAP/TAZ expression is associated with poor prognosis in hepatocellular carcinoma (HCC), cholangiocarcinoma patients, lung, and colorectal cancers [65,66,67,68]. Moreover, high YAP/TAZ expression in cancer may also predict resistance to therapies and cancer relapse [69,70]. Taken together, these results demonstrate that Hippo pathway, especially YAP/TAZ activity, is involved in human cancer development and may function as a molecular target for cancer diagnosis and therapy.

Even though activation of YAP/TAZ occurs frequently in human cancers, the mutation rates of Hippo pathway genes are unexpectedly modest. However, one exception is *NF2*. Inactivating mutation of *NF2* is observed in multiple cancers including meningiomas, schwannomas, and mesotheliomas [71,72]. Additional genetic alterations of Hippo pathway genes in cancer have also been reported, for instance, WWTR1-CAMTA and YAP1-TFE3 fusion are found in epithelioid hemangioma [73,74]. In addition, silencing of *MST1/2*, *LATS1/2*, or *RASSF1* due to promotor hypermethylation are reported in soft tissue sarcomas, breast cancer, and lower-grade glioma [20,75,76,77,78,79]. However, these genetic or epigenetic alterations are not sufficient to explain the widespread YAP/TAZ activation in cancers, especially in cancers with high incidences, and additional molecular mechanisms may contribute to YAP/TAZ activation in cancer.

## 3. Regulation of Hippo Pathway by GPCRs

GPCRs represent the largest family of cell surface receptors in human genome, and they are involved in a wide range of physiological processes by transmitting diverse extracellular signals into cells. Recent studies suggest that the Hippo pathway is a downstream branch of GPCR signaling. Many GPCRs mediated signals can modulate YAP/TAZ activity, either positively or negatively, dependent on the nature of signals, receptors, and adaptor proteins [4,31].

Following the initial discovery that sphingosine-1-phosphate (S1P) and lysophosphatidic acid (LPA) can induce YAP/TAZ activity, diverse GPCR related signals have been shown to modulate YAP/TAZ activity [30,32] (Table 1). For instance, simple molecules such as protons, which are associated with extracellular pH, can induce YAP activity [80]. Metabolites such purines, adenosine, epinephrine, glutamate, fatty acids, and bile acids activate YAP through GPCRs stimulation [30,32,81,82,83]. Polypeptides and secreting proteins, such as thrombin, glucagon, Angiotensin II, and Endothelin, also modulate YAP/TAZ activity via GPCR signaling [32,84,85,86,87]. These signals, either locally or from a long range, represent major constitutes of cell niche or microenvironment, suggesting that the Hippo pathway is regulated collectively by signals surrounding a given cell. Adhesion GPCRs link cells to their neighbors and probably cell matrix [88], these receptors may also link physical signals to the Hippo pathway. 

**Figure 1:**
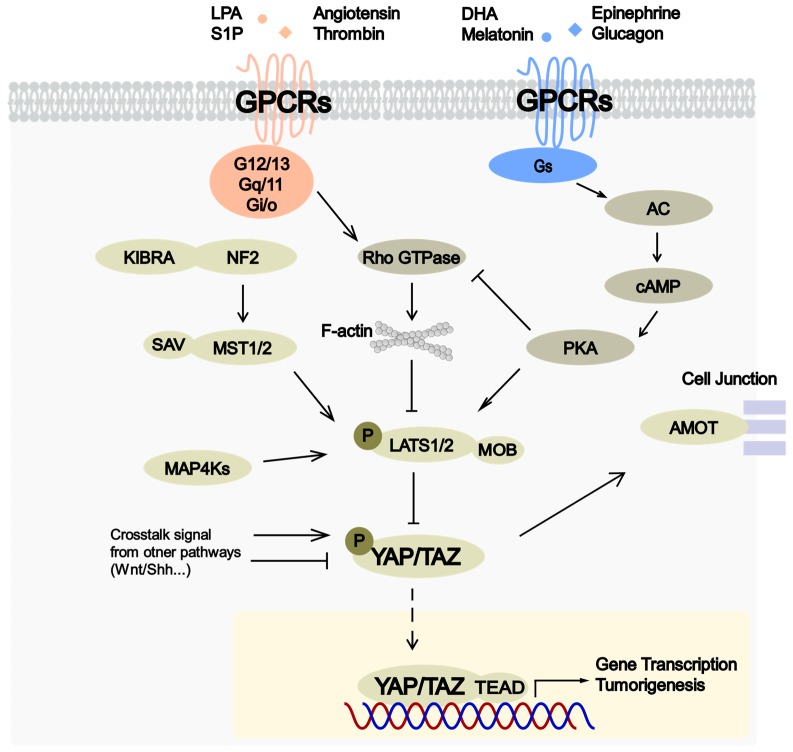
GPCRs modulate Hippo-YAP signaling via GPCRs–G-protein–cytoskeleton axis. The molecular scheme of GPCR-Hippo signaling is shown, including activating and inhibitory regulation of YAP/TAZ. GPCRs and G proteins activating YAP/TAZ are marked with red and those inhibiting YAP/TAZ are in blue.

GPCR ligands regulate the Hippo pathway differentially (Figure 1). It has been established that the effect of GPCR ligands on YAP/TAZ activity is dependent on the type of downstream G proteins activated [32]. GPCRs coupled with Gα_12/13_, Gα_q/11_, or Gα_i/o_, such as LPA and thrombin receptors, will activate YAP/TAZ; on the contrary, GPCRs coupled with Gα_s_, such as epinephrine and glucagon receptors, will inhibit YAP/TAZ [32]. The function of GPCRs and G proteins on the Hippo signaling is most likely depended on protein kinases (such as PKA and PKC), Rho GTPases, and remodeling of the actin cytoskeleton [4]. PKA has been proposed to mediate upstream signals by repressing actin fiber formation or phosphorylating LATS1/2 directly [107,108,109]. The effect of PKC appears to be isoform-specific, for instance canonical PKC isoforms induce YAP/TAZ activity, whereas novel PKC isoforms repress YAP/TAZ activity [110]. The isoform-specific effect of PKC towards YAP/TAZ may explain cell type-dependent response to PKC activation, as the expression of PKC isoforms vary across different cell types. It seems like MST1/2 is not a direct target of GPCR signaling but the phosphorylation level of LATS1/2 is sensitive to different GPCR ligands [32]. In the absence of MST1/2, MAP4Ks may be responsible for LATS1/2 phosphorylation, as the deletion of MST1/2 and MAP4Ks together abolished the regulation of LATS1/2 phosphorylation by GPCR signaling [5]. Collectively, G proteins and related kinases relay the GPCR signaling to regulate dynamics of the actin cytoskeleton, which, in turn, can be sensed by the Hippo pathway. How different states of actin cytoskeleton sensed by Hippo pathway components remains unclear.

Some seven-(pass)-transmembrane domain receptors, such as Frizzled and smoothened (SMO), are not considered as typical GPCRs. Recent evidence suggests that these atypical GPCRs also regulate the Hippo pathway in a G protein-dependent manner. For instance, Wnt ligands and their receptors (Frizzle proteins) can repress LATS1/2 activity and lead to enhanced YAP/TAZ activity [111]. In addition, Hedgehog (Hh) ligands, via SMO -Gα_s_-cAMP-PKA signaling axis, lead to repression of YAP/TAZ [112]. Thus, atypical GPCRs can regulate the Hippo pathway and also contribute to the crosstalk between Hippo and other important pathways (such as Wnt and Hedgehog) in development and cancer.

The regulation of Hippo pathway by GPCR signaling can also be fine-tuned by additional signals. For instance, it has been shown that the effect of GPCR on YAP/TAZ activity is enhanced when insulin is present, and PI3K and PKD downstream of insulin receptor are involved in this regulation [113]. Moreover, MAPK signaling has also been shown to modulate the Hippo pathway [114]. Hence, the crosstalk between the GPCR-Hippo signaling axis with other pathways should be explored in the future.

## 4. Widespread Alternations of GPCR-YAP Signaling Axis in Cancer

Aberrant GPCR signaling is an important mechanism in cancer development [115]. Different effectors, such as MAPK signaling, mediate the aberrant GPCR signaling to promote tumorigenesis [116]. As a new downstream branch of GPCR signaling, the Hippo tumor suppressor pathway may also play a role in this process. Moreover, aberrant GPCR signaling represents a potential mechanism responsible for prevalent YAP/TAZ activation in human cancers (Table 1). 

Cancer genome sequencing analyses revealed that mutations in GPCRs and G proteins are widespread and frequent in multiple tumor types [117]. It has been reported that *GNAQ* and *GNA11* encoding the alpha subunits of Gα_q_ and Gα_11_, respectively, are frequently mutated at Arg183 or Gly209 in uveal melanoma and blue nevi [118,119], the mutations at Arg183 and Gly209 result in constitutive activation of Gα_q/11_, and several downstream effectors including YAP/TAZ are activated, leading to tumorigenesis [120,121]. Mutations in *GNAS* has been discovered in human medulloblastoma [122] and it has been demonstrated in mice that *GNAS* loss leads to YAP activation and tumorigenesis [109,123]. Thus, YAP activation might be a common mechanism underlying cancer associated mutations on G proteins. However, mutations at Gly227 on *GNAS* contribute to the development of hormone-secreting pituitary tumors and thyroid adenomas [124,125], in principle this mutation will inactivate YAP/TAZ [126].

Mutations in genes encoding GPCRs are observed in approximately 20% of cancers, including mutations in *TSHR* in thyroid cancer, luteinizing hormone receptor (*LHCGR*), and follicle stimulating hormone receptor (*FSHR*) in breast, lung, and colon cancers [127]. Smoothened (*SMO*) is also frequently mutated in cancers arising at the colon, central nervous system, and many other cancers types [128]. Mutations observed in the family of GPCR adhesion receptors, the majority of which are still orphan, resulted in constitutive activation of the receptors, leading to pathological conditions [129]. Moreover, mutated glutamate receptors, such as GRM8, GRM1, and GRM3, have been implicated in squamous non-small cell lung cancer (NSCLC) and melanomas [126]. Currently, the effect of these mutations on the Hippo pathway has not been systematically examined.

Aberrant expression of GPCRs and their ligands may also contribute to tumorigenesis. Elevated *PAR1* (a thrombin receptor) expression is associated with poor differentiation and metastasis of breast cancer [130]. Increased expression of G protein–coupled estrogen receptors (*GPER*) and TAZ activation are detected at the early stage of breast tumor development [89]. Additionally, aberrant expression of LPA receptors may elicit cancer initiation and progression in breast cancer and ovarian cancer via activation of the Rho-dependent transduction pathway [131]. Abnormal G-protein coupled hormone receptor also involved in several adrenal diseases, including tumor and hyperplasia [132]. Kaposi’s sarcoma (KS) is caused by infection of human herpesvirus 8 (HHV-8, also known as KSHV) and a viral GPCR (vGPCR) encoded in HHV-8 genome can activate TAZ, which is essential for the development of KS [133]. Recently, an orphan G protein-coupled receptor GPRC5A has been identified as a hypoxia-induced protein, which protects hypoxic tumor cells from apoptosis via the HIF-GPRC5A-RhoA-YAP axis [134]. For GPCR ligands, the levels of LPA has been considered as a marker for ovarian cancer, and high circulating angiotensin II is associated with carcinogenesis, prognosis, and drug resistance in several malignancies, such as colorectal cancer, hepatocellular carcinoma, melanoma, ovarian cancer, and breast cancer [135,136,137,138,139,140,141]. Together, abnormal expression of GPCRs and GPCR ligands are associated with the development of different cancers and YAP/TAZ activation may, at least in part, participate in the tumorigenic process.

## 5. Potential Cancer Therapies Targeting GPCR-Hippo Signaling Axis

Given the frequent dysregulation of GPCR signaling and Hippo pathway in cancer, the GPCR-Hippo signaling axis may serve as a therapeutic target for cancer treatment. As the Hippo pathway is a downstream branch of the GPCR signaling, cancers initiated by aberrant GPCR signaling might be treated by modulating the Hippo pathway, especially YAP/TAZ activity. Meanwhile, for cancers with a dependency on high YAP/TAZ activity, drugs targeting GPCRs and G proteins may reduce YAP/TAZ activation and slowdown cancer progression.

The Hippo pathway can be modulated at multiple levels [69]. As the function of Hippo pathway is mediated mainly by YAP/TAZ and associated transcription factors TEAD1-4, different approaches have been developed to disrupt YAP/TAZ-TEAD interaction. Liu and colleagues discovered the porphyrin family compounds, such as verteporfin (VP), can effectively block YAP-TEAD interaction [142]. A VGLL4-mimicking peptide has also been developed to displace YAP from TEAD [61]. Recently, it has been reported that the pocket in TEAD critical for YAP/TAZ binding is palmitoylated and targeting TEAD palmitoylation is a new approach to repress YAP/TAZ activity [143,144,145,146]. Both VP and VGLL4-mimicking peptides have been used in vitro and in vivo to repress YAP/TAZ activity, tissue growth, and tumorigenesis, whereas further improvement of these drugs might be required for clinical use.

GPCRs represent a major target of currently available drugs, and some GPCR-based drugs could be repositioned to block YAP/TAZ activity. For instance, Gα_s_-targeted molecules may repress YAP/TAZ activity in a way that is similar to epinephrine, dobutamine, and glucagon [106,147]. In contrast, antagonizing or depleting Gα_12/13_-, Gα_q/11_-, or Gα_i/o_-mediated signals, such as using phosphatase-resistant LPA analogues and monoclonal antibodies specific for LPA or S1P [117,148], may also limit YAP/TAZ activity. It is noteworthy that some GPCR-based drugs, like β-blockers and dopamine, will significantly affect heart and psychiatric functions, thus, side effects must be considered before using these drugs in cancer therapies [149,150].

Proteins transmitting GPCR signaling can also be targeted to manipulate the Hippo pathway. Recently, cyclic depsipeptide FR900359 has been shown to target mutant Gα_q/11_ and repress downstream effectors MAPK and YAP [151,152]. The activity of PKA is associated with cellular cyclic-AMP (cAMP) levels, Forskolin or phosphodiesterase inhibitors, such as Rolipram, have been shown to induce PKA activity and repress YAP/TAZ [112,148]. PKC inhibitors can also repress YAP/TAZ activity in a cell type-dependent manner [121]. Rho GTPases are central for the regulation of Hippo pathway by GPCR signaling, it has been shown that statins, inhibitors of HMG-CoA reductase (HMGCR), can indirectly inactivate Rho GTPases and reduce YAP/TAZ nuclear localization [153,154]. As GPCR-Hippo signaling is a complex signaling network, drugs targeting GPCRs, G proteins, or downstream signaling nodes may affect effectors other than YAP/TAZ, hence, the specificity towards the Hippo pathway will be compromised when using these drugs.

## 6. Conclusions

In the last few years, Hippo pathway has been under the spotlight due to its function in organ size control and tumorigenesis. GPCRs, the largest cell membrane receptor family, regulate an array of downstream signal pathways, including Hippo pathway effectors YAP/TAZ. Aberrant GPCR and YAP/TAZ activation have been observed in pathogenesis of several types of cancer. Although further in-depth studies are still required to address issues relevant to side effects and pharmacodynamics, it is clear that GPCR-Hippo signaling axis will be a promising target for anticancer therapy.

## Figures and Tables

**Table 1:** The regulation of YAP/TAZ activity by various G-protein coupled receptors in human cancers. Representative GPCRs that are most frequently implicated in human cancer are shown and their regulation on YAP/TAZ activation are listed in the following table.

GPCRs	Ligand	Coupling Protein	YAP/TAZ Activation	Associated Cancer Type	References
GPER	Estrogen	Gαq/11	↑	Breast cancer	[89]
LPA receptors	LPA	Gα12/13, Gαq/11	↑	Colon cancer Ovarian cancer Prostate cancer Breast caner	[32,90]
S1P receptors	S1P	Gα12/13	↑	Hepatocellular carcinoma	[91]
Protease-activated receptors (PARs)	Thrombin	Gαq, Gα12/13, Gαi	↑	Melanoma Colon cancer Breast cancer Lung cancer Pancreatic cancer Prostate cancer Squamous cell carcinoma of the head and neck	[87,92,93]
ETAR	Endothelin-1	Gαq/11	↑	Colorectal cancer	[86]
EP2, EP4	PGE2	Gαq/11	↑	Colon caner Hepatocellular Carcinoma Head and neck cancer Non-small-cell lung cancer	[94,95]
Frizzleds (FZD)	Wnts	Gα12/13	↑	Colorectal cancer Prostate cancer Hepatocellular carcinoma	[96]
Chemokine (C-X-C motif) receptor 4	SDF1/CXCL12	Gα12/13, Gαq/11, Gαi/o	↑	Breast cancer Non-small cell and small cell lung cancer Oral squamous carcinoma Chronic Myelogenous Leukemia	[97,98,99]
Chemokine (C-X-C motif) receptor 2	IL8, CXCL5	Gαi	↑	Head and neck squamous cell carcinoma Non-small cell lung cancer (NSCLC) Ovarian cancer	[100,101,102]
Angiotensin II receptor AT1	Angiotensin II	Gαq/11	↑	Prostate cancer Cholangiocarcinoma	[103,104]
Free Fatty Acid receptor 1(FFAR1)	Fatty acids	Gαq/11, Gαi/o	↑	Prostate cancer	[105]
β1- and β2-adrenergic receptors	Catecholamines (e.g., dobutamine)	Gαs	↓	Breast cancer	[106]
